# Neurophysiological Assessment of Prolonged Recovery From Neuromuscular Blockade in the Neonatal Intensive Care Unit

**DOI:** 10.3389/fped.2020.00580

**Published:** 2020-09-18

**Authors:** Omri David Soffer, Angela Kim, Ellen Underwood, Anne Hansen, Laura Cornelissen, Charles Berde

**Affiliations:** ^1^Division of Newborn Medicine, Boston Children's Hospital, Boston, MA, United States; ^2^Department of Paediatrics, Harvard Medical School, Boston, MA, United States; ^3^Department of Anesthesiology, Critical Care & Pain Medicine, Boston Children's Hospital, Boston, MA, United States; ^4^Department of Anaesthesia, Harvard Medical School, Boston, MA, United States

**Keywords:** neuromuscular relaxants, rocuronium, vecuronium, neonate, infant, children, train-of-four (TOF)

## Abstract

**Objective:** To evaluate recovery from neuromuscular blockade in infants using Train-of-Four nerve stimulation.

**Study Design:** Ulnar nerve stimulation was used to evoke thumb twitch and reported as Train-of-Four ratio. Thumb twitch was also recorded visually in real-time. Primary outcome was time to near recovery of muscle function (Train-of-Four ratio >70%). Secondary analyses were time to greater degrees of recovery (Train-of-Four ratio >80, 90%), sensitivity of accelerometry vs. visual thumb-twitch and clinical variates to assess safety.

**Results:** Patients were enrolled following rocuronium-boluses (*n* = 10) and vecuronium-infusions (*n* = 9). Median recovery time to Train-of-Four ratio >70% was 14 h following rocuronium-bolus dosing and 34 h following cessation of continuous vecuronium infusion. Median stimulus threshold for accelerometry was 27.5 mA and visual observation was 20 mA. There were no safety concerns.

**Conclusion(s):** Neuromuscular monitoring using Train-of-Four nerve stimulation is feasible in infants. Some infants exhibited prolonged recovery from neuromuscular-blockade. These pilot data may facilitate future standardized pediatric protocols on neuromuscular monitoring for safer dosing.

## Introduction

Neuromuscular blocking agents (NMBA) are widely administered in infants by bolus dosing to permit muscle relaxation during surgery or to facilitate tracheal intubation. They are also administered as a prolonged continuous infusion in intensive care units (ICUs) to minimize movement, decrease oxygen consumption, and facilitate procedures and mechanical ventilation. In adults and older children in the operating room (OR), depth of neuromuscular block (NMB) is commonly measured using peripheral nerve stimulation, yet this valuable practice is inconsistently applied to infants either in the OR or ICU setting (1, 2).

The most common means by which NMB level is quantitatively monitored is a technique called Train of Four (TOF) in which electrical stimuli are delivered to a peripheral nerve to elicit motor responses (twitches) in the corresponding muscle. The strength of contraction indicates the degree of recovery from NMB.

The TOF pattern of twitch stimulation was developed in 1970 to provide an easily applicable semi-quantitative tool to assess NMB in clinical settings (3). The rationale was to produce a pattern of stimulation that did not require measurement of absolute magnitudes of evoked responses or comparisons to control responses obtained before administration of a neuromuscular blocking drug. A series of 4 identical electrical stimuli are applied to a peripheral nerve at intervals of 0.5 s (2 Hz) and motor responses (twitches) are observed in the muscles innervated by that nerve. Most commonly, this is applied to the ulnar nerve to evoke thumb adduction due to adductor pollicis contraction. In routine clinical practice, visual responses are observed. In clinical pharmacology studies, responses can be quantified by recording acceleration of the thumb or other body part. In the absence of NMB, these 4 twitches are of equal amplitude. In the presence NMB from non-depolarizing drugs, repeated stimuli induce progressively weaker responses (twitch fade), which is reflected in reductions in the ratio of the magnitude of the fourth twitch (T4) compared to the first twitch (T1). This is called the TOF ratio.

When a non-depolarizing NMBA is given, a typical pattern is observed. T4 disappears followed by T3, T2, and finally T1. The reverse is true during recovery from non-depolarizing block: T1 reappears first followed by T2, T3, and finally, T4. The most common clinical application of the TOF ratio is in monitoring recovery from NMB at the end of surgery. Traditionally, it had been accepted that a TOF ratio of 70% or greater was an indication of adequate NMB recovery. More recent adult studies favor recovery to TOF ratio 90% or greater prior to extubation after surgery, whether achieved by spontaneous recovery or via neuromuscular block reversal agents, based on the observation that postoperative respiratory complications in adults may be associated with incomplete degrees of neuromuscular recovery (1, 3). Residual muscular blockade also results in risk of airway obstruction, and in a decreased response to hypoxemia (4).

Surveys of practice patterns indicate that TOF is rarely used in the NICU or Pediatric ICU during prolonged infusions (2, 5). Instead, subjective clinical assessment is the most common means of determining degree of NMB (2). The lack of validated standards or guidelines in the NICU raises the concern for inadequate control of the degree of chemical paralysis.

The limitations of subjective assessment and under-recognition of residual paralysis may be a particular concern with use of NMBA among infants. Skeletal muscle and the diaphragm undergo extensive functional maturation during the first year of life (6). The neonatal diaphragm lacks fatigue-resistant type 2 fibers, and neonates have multiple physiologic factors that increase vulnerability to respiratory failure (7). For many medications, dose-response and duration vary greatly in early infancy due to pharmacokinetic and pharmacodynamic maturation (8, 9), and can also be affected by co-administration of other medications, such as antibiotics and corticosteroids (10). Because of the variability in dose response, monitoring of neuromuscular function while administering a continuous infusion of an NMBA in the NICU may help to ensure adequate, but not excessive, chemical paralysis.

Our overall aim is to quantitatively evaluate NMB recovery in neonates receiving NMBA via bolus dosing for surgery and prolonged infusions in the NICU. Thereby we aim to provide valuable pilot information to foster further clinical outcomes and safety studies of NMB administration in infants in the NICU.

## Materials and Methods

### Study Design

**Figure 1:**
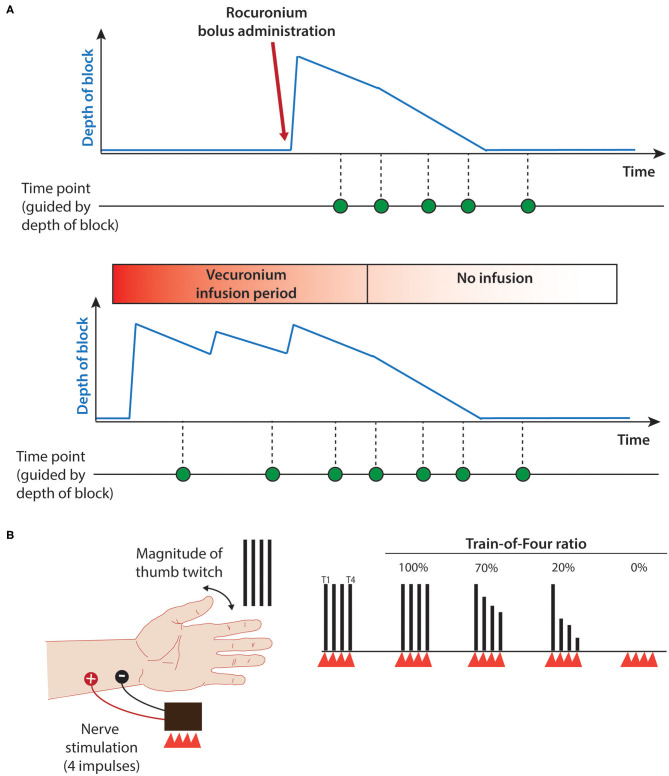
Study protocol. **(A)** Schematic of study timeline for rocuronium bolus (upper panel) and continuous vecuronium infusion (lower panel). Blue line provides estimate of depth of block. Green dots indicate potential data collection time-point. **(B)** Train-of-Four assessment using ulnar nerve stimulation. Red triangles indicate train of four electrical stimuli; black vertical lines indicate degree of stimulus-evoked thumb twitch. T1, first stimulus; T4, fourth stimulus; Train-of-four ratio, T1/T4. 100% indicates complete nerve block whereas 0% indicates complete neuromuscular recovery. Adapted from (11).

This is an observational, open-label, single-site pilot study investigating recovery from NMB in infants aged 0–6 months chronologic age (Figure 1). Our hypotheses are: (1) infants exhibit prolonged residual blockade following rocuronium bolus administration or cessation of continuous vecuronium infusions, and (2) acceleromyography is a feasible method to assess muscle function in infants.

### Participants

Between March 07, 2019 and January 19, 2020, infants admitted to the Boston Children's Hospital Neonatal Intensive Care Unit (Boston, Massachusetts, USA) were screened for eligibility and enrolled in the study.

Infants were eligible for inclusion if they were at least 25 weeks' gestational age and <6 months chronologic age with a planned or ongoing exposure to rocuronium or vecuronium. Infants were excluded in case of (1) confirmed or suspected congenital neuromuscular dysfunction or (2) circumstance that would interfere with conducting the study (e.g., IV access or skin lesions over the intended site for electrode placement, musculoskeletal abnormality of the upper extremities). All infants were assessed for eligibility by a single physician (ODS).

### Ethical Approval

This study was reviewed by the Committee on Clinical Investigation at Boston Children's Hospital and approved by The Institutional Review Board (IRB) review under federal regulation 45 CFR 46.101(b) (IRB P00029902, date approved: 12/7/2018). A written informed parental consent was obtained for all infants.

### Study Time-Points

TOF ratios and visual thumb twitch were recorded when feasible during steady state infusions, daily interruptions of vecuronium infusions (i.e., “vecuronium holiday”), and after cessation of vecuronium infusions or rocuronium boluses (Figure 1A).

Two distinct patient groups were studied: Preterm and term neonates and young infants (A) in the OR and NICU with resolving effects of intraoperative bolus dosing of rocuronium given during and after thoracic, abdominal and pelvic surgical procedures, and (B) receiving prolonged vecuronium infusions, including a unique population of infants with long-gap esophageal atresia undergoing an esophageal growth induction procedure who can require weeks of immobility while tension is placed on the upper and/or lower esophageal pouches. Other clinical indications for vecuronium infusion were severe pulmonary hypertension or post-operative tracheostomy care.

All decisions regarding dosing of NMBAs were made by clinicians according their usual practice; no aspect of dosing was modified for purposes of this study.

### Demographics and Medical History

Demographic data and medical history including age, weight, clinical diagnoses, and surgical procedures were obtained from the electronic medical record.

### Thumb-Twitch Paradigm

#### Paradigm

We used a peripheral nerve stimulator device and a tri-axial accelerometer to monitor NMB (StimPod NMS 450, Xavant Technologies, South Africa). The procedure was performed by electrical stimulation of the ulnar nerve, which innervates the adductor pollicis muscle with the accelerometer placed over the thumb. Four electrical stimuli separated by 0.5 s interstimulus-interval were applied to the ulnar nerve just above the wrist using the TOF device (Figure 1B).

All infants were receiving opioids and sedatives at the time TOF measurements were performed. The intensity of the peripheral nerve stimulus was titrated from 5 mA to a maximum of 80 mA in increments of 5 mA until a relatively constant TOF ratio was achieved, termed the “supramaximal stimulus.” If no twitches were observed stimulation stopped at 50 mA. Each train was performed three times every 15 s before moving up to the next stimulus intensity.

Pre-determined criteria to discontinue the measurement were increased heart rate by 20 beats per minute and/or decreased oxygen saturation level over 10% during the elicited stimulus due to plausible concern of discomfort. Heart rate and oxygen saturation were recorded during 2-min baseline periods prior to the start of stimulation and during the TOF stimulation. After each study, the skin in the vicinity of the surface electrodes was inspected for any lesions. Should any safety concerns arise, the study would be terminated.

#### Peripheral Nerve Stimulator Device

The StimPod device includes a nerve mapping probe, a tri-axial accelerometer and a handheld display monitor. The mapping probe is used to ensure the precise location of the ulnar nerve prior to starting the study. The accelerometer provides real time feedback of the strength of contraction of the affected limb. The accelerometer measures ~22 mm × 28 mm × 10 mm (width × length × depth), and combined weight including the manufacturer's plastic thumb attachment strap is 5 g. The display monitor provides a read-out of the force of each contraction and the TOF ratio.

The advantages of using the StimPod device are that (1) the accelerometer calculates the movement vector of the contraction in three dimensions (*x, y, z*) which excludes the need for calibration prior -unlike one-dimensional accelerometers; (2) age-appropriate sized electrodes (i.e., pediatric leads used here) can be used; (3) nerve mapping can be used in very young patients; and (4) overall set-up time of the procedure is relatively quick. The StimPod device is also widely used and commercially available.

### Thumb-Twitch Assessment

#### Study Set-Up

Pediatric-sized hydrogel electrodes (22.2 mm square, NeuroPlus™ cloth snap-electrode series, Buffalo, NY) were positioned over the ulnar nerve at the wrist. The StimPod accelerometer was attached to the thumb using the manufacturer's plastic strap. Prior to the start of the TOF paradigm, a StimPod nerve mapping probe was used to ensure precise location of the ulnar nerve. Movement of the thumb and hand were not restricted.

The thumb-twitch assessments were performed at the bedside by a neonatology fellow (ODS). Muscle response after each stimulus train was observed. Two methods of assessment were performed:

1. Visual thumb twitching. A subjective method, which evaluates the number of twitches by seeing presence of thumb twitch in real-time at the bedside.
2. Accelerometry. A quantitative method, which records the force of contraction made by the thumb upon each of four twitches and calculates the TOF ratio.

The assessor recorded the visual response on a data collection sheet, followed by the accelerometry ratio read-out from the StimPod display. The assessor was unblinded to the results of the motor response and the accelerometry output.

### Outcome Measures

The primary outcome measure was time-to-recovery of muscle function (TOF ratio >70%). Secondary analyses compared cumulative time to various degrees of recovery (TOF ratio >80 and 90%), sensitivity of accelerometry compared to visual detection of thumb twitch, and clinical safety variables consisting of change in heart rate and oxygen saturation between baseline and during stimulation. TOF ratio >70% was chosen as primary outcome variable instead of >80% or >90% due to the anticipation of very slow or incomplete recovery in a subset of patients.

### Statistical Methods

#### Sample Size

As an observational, non-interventional pilot study, this was a sample of convenience.

#### Statistical Analysis

Statistical analysis was performed using the GraphPad Prism 8.0 (GraphPad Software, Inc., San Diego, CA). Demographic, clinical characteristics, TOF ratios above 80 and 90% were summarized using descriptive statistics either by means (SD), median (IQR) or range according to whether the data were normally distributed or skewed and percentages were given for categorical variables. Normality testing was performed with D'Agostino-Pearson test.

Accelerometry measurement is reported as TOF ratio at corresponding stimulus amplitudes elicited by the peripheral nerve stimulator. Each measurement was repeated 3 times and median TOF ratio was calculated.

Comparison between stimulus amplitude thresholds to elicit visual thumb twitch and acceleromyography output was reported using a paired non-parametric Wilcoxon signed rank test. Spearman Simple linear regression was used to study the correlation between the current weight of the participants to the stimulus threshold (mA) required for eliciting visual thumb twitch and accelerometry measurement.

Kaplan–Meier curves were used to illustrate time course of NMB recovery (TOF ratio >70%) for each study group. Infants whose TOF ratio was not 70% or greater were censored. A *p* < 0.05 was considered statistically significant.

## Results

TOF stimuli were delivered to the ulnar nerve following either rocuronium boluses or cessation of vecuronium infusions in 17 infants aged 0–6 months chronologic age. Fifteen infants were studied on multiple occasions (range 2–5 measurements for each participant). Ten infants were in the rocuronium-bolus group, and 9 infants were in the vecuronium-infusion group; Two infants were included in both groups. Infant demographics and clinical characteristics are found in Table 1.

**Table 1:** Demographics and relevant clinical history.

	**All (*n* = 17)**	**Rocuronium (*n* = 10)^a^**	**Vecuronium (*n* = 9)**
**Demographics**			
Male (*n*, %)	8 (47%)	4 (40%)	6 (67%)
Gestational age in weeks	34 (±4.3)	35 (±2)	33 (±5.4)
PMA at study in weeks (median)	42 (37.4–47.5)	40 (37–44)	43.5 (38.8–50.4)
Chronological age in days (median)	63 (7–119)	11 (6.8–80)	82 (19.5–148)
Birth weight in kg	2.2 (±1)	2.58 (±0.9)	1.8 (±0.9)
Weight at study in kg	4.1 (±1.6)	3.63 (±1.3)	4.6 (±1.8)
**Neuromuscular block medication**			
Indication (*n*, %)			
Esophageal atresia repair	8 (43%)		
Abdominal surgery	4 (23%)		
Airway surgery	3 (17%)		
Other^b^	3 (17%)		
Dose at study time point (median)	–	1.5 (1.2–1.5) mg/kg/dose	0.1 (0.1–0.11) mg/kg/h
Duration of infusion in hours (median)	–	–	172 (77.5–323)

*Data given as mean unless otherwise stated. Interquartile ranges are given for each median*.

a *Two infants were included in both the rocuronium bolus dosing group and the vecuronium prolong infusion group*.

b *Neuromuscular block indications: “Other” include pulmonary hypertension, spinal mass removal, and post-operative tracheostomy care*.

### Baseline and Clinical Characteristics

Baseline characteristics were similar between the two groups (Table 1). Vecuronium infusion rates were maintained within a narrow range (0.1–0.11 mg/kg/h). The duration of vecuronium infusion varied considerably (~2–18 days), based on clinical indications.

### Neuromonitoring Measures in Infants

We reviewed the optimal parameters required for neuromuscular monitoring in infants in this pilot study, namely (1) Identification of thumb twitch; and (2) TOF stimulus intensity.

### TOF Stimulus Intensity

All infants eventually demonstrated a thumb twitch following cessation of NMBA administration. The median stimulus intensity required to evoke a thumb twitch was 20 mA, ranging from 10 to 45. The youngest infant was 2 days old and responded to stimulus intensity of 20 mA following rocuronium bolus dosing. The lightest infant weighed 2.12 kg and also responded to a stimulus intensity of 20 mA.

Regarding the pre-determined safety parameters, no infant had a change in heart rate or oxygen saturation between baseline recording period and stimulus delivery which would have resulted in discontinuation of the TOF measurement. Following removal of stimulation electrodes from the wrist, some patients demonstrated mild transient erythema, but in no cases was it clinically significant or persistent.

### Assessment of Thumb Twitch

**Figure 2:**
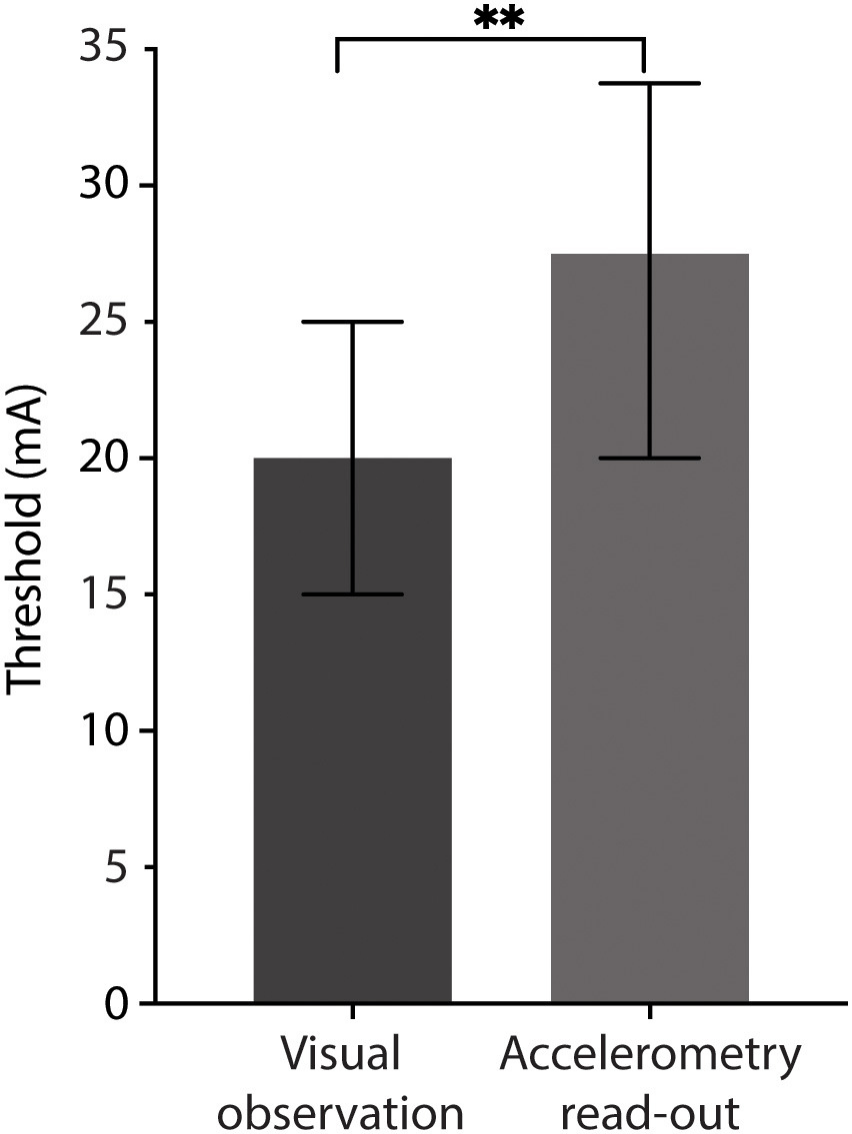
Stimulus threshold required to elicit visual thumb twitch and accelerometry. Median stimulus thresholds for visual thumb twitch and accelerometry read-out. ^**^p < 0.01, unpaired Wilcoxon test.

Threshold current for evoking thumb twitch by visual assessment and by accelerometry were evaluated upon cessation of NMBA administration. Median stimulus amplitude threshold of the 17 infants for a visual thumb twitch was 20 mA (interquartile range 15–25) and 27.5 mA for accelerometry (interquartile range 20–33.8); Figure 2. This difference was statistically significant (Wilcoxon signed rank test *p* < 0.01) but a clinically minor effect size.

**Figure 3:**
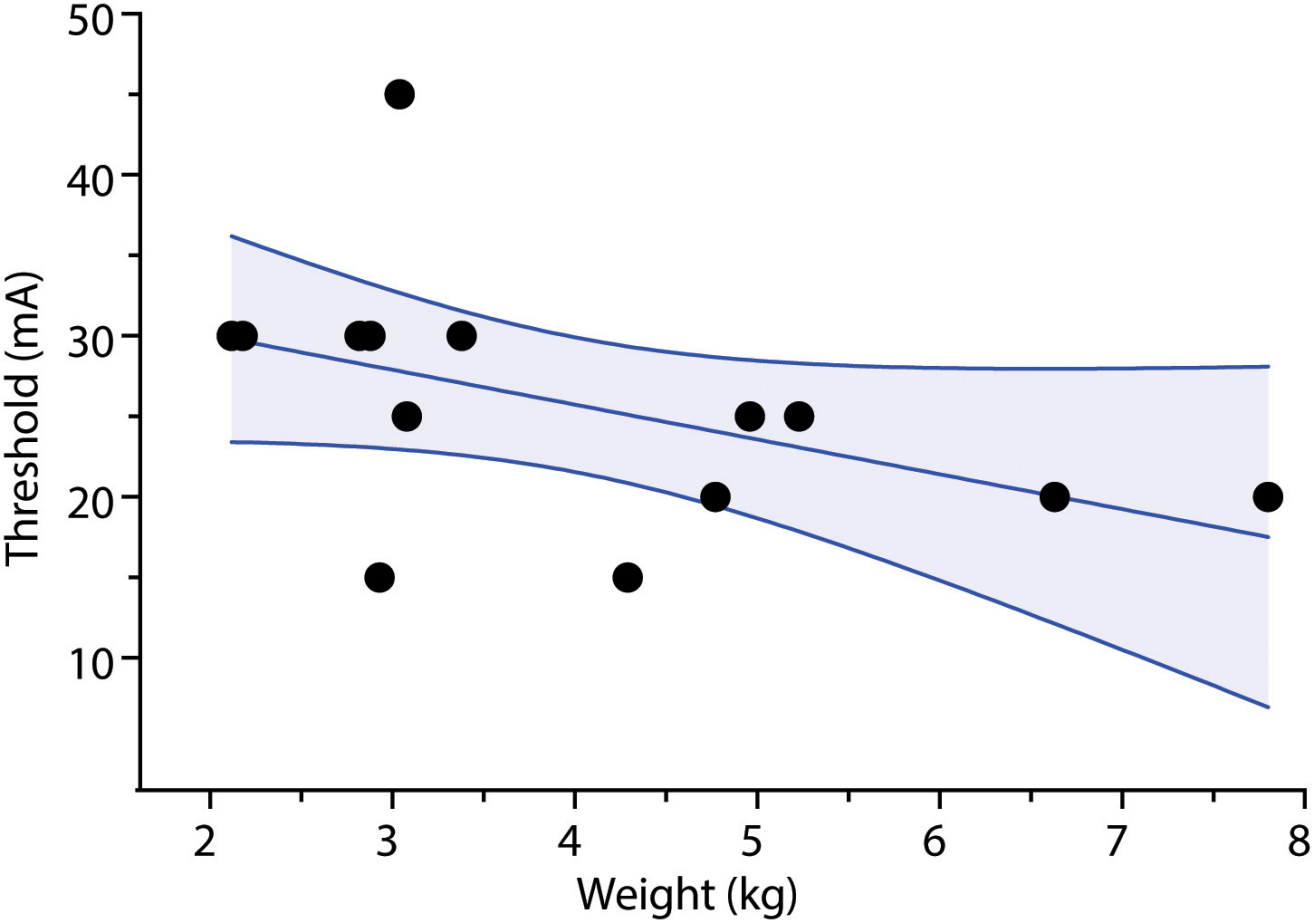
Correlation of stimulus threshold for accelerometry measurement and weight. Stimulus threshold for accelerometry measurement output is negatively correlated with body weight. *p* < 0.05, Spearman's correlation test *r* = −0.56. Dots represent stimulus threshold required to elicit a train-of-four accelerometry read-out in individual infants. Solid line represents line-of-best-fit; shaded area represents 95% confidence interval limits.

A slight negative correlation was noted between body weight at time of study and the stimulus threshold needed for an accelerometry measurement for infants recovering from NMB as shown in Figure 3 (*r* = −0.5, *p* = 0.03).

**Figure 4:**
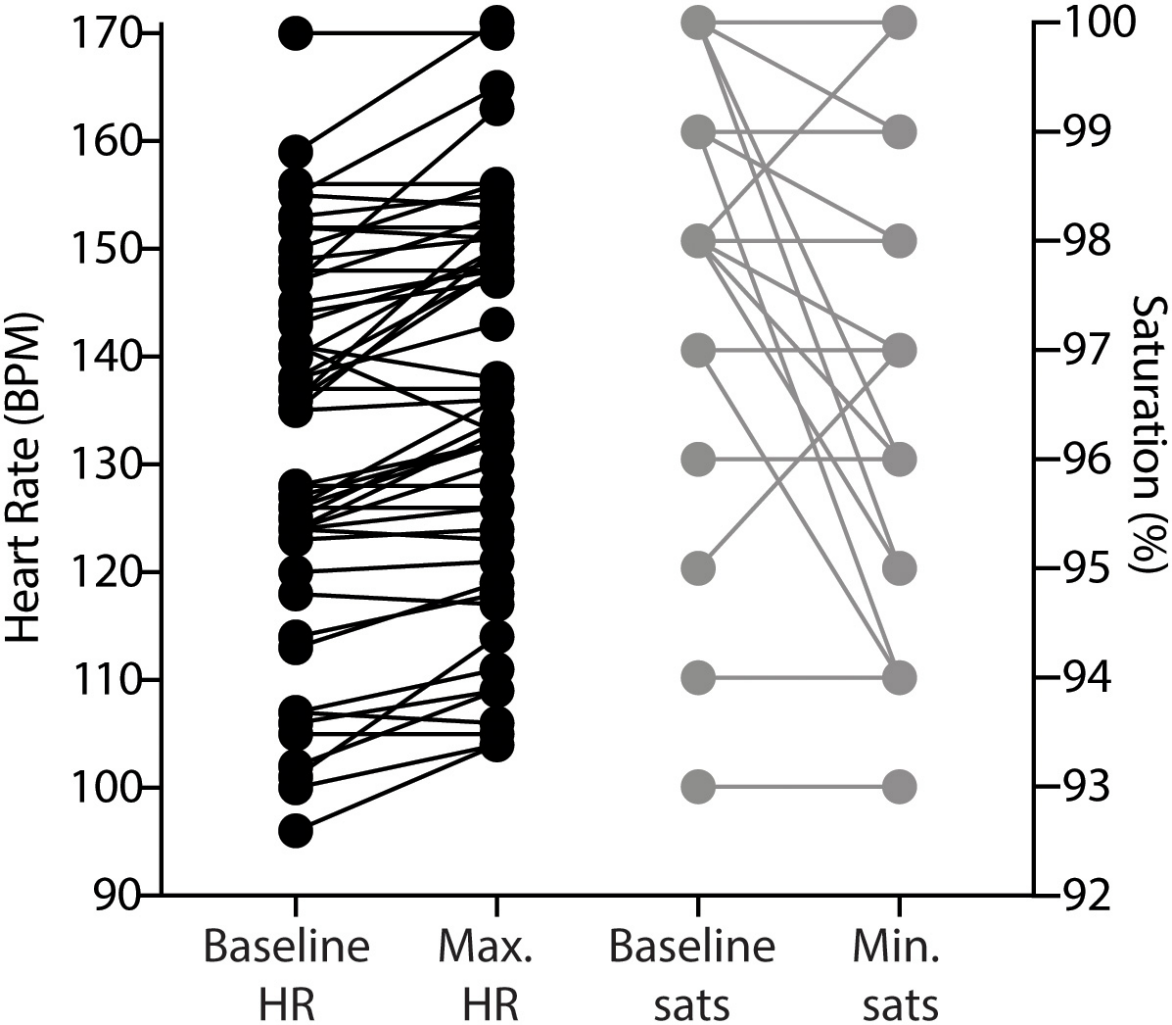
Change in heart rate and oxygen saturation during accelerometry measurements. Dots indicate individual trial read-outs; lines indicate within-trial measurement. BPM, beats per minute; HR, heart rate; sats., oxygen saturation.

At the highest stimulation intensity for each infant, compared to resting baseline, there was a statistically significant, but clinically minor increase in heart rate and reduction in oxygen saturation following TOF stimulation. The mean difference between the baseline heart rate of infants to peak heart rate during stimulation of TOF in both the rocuronium bolus dosing group and in the vecuronium continuous infusion group was 4.5 BPM (+5.5 SD, *p* < 0.05). The mean difference between the baseline saturation level to minimal saturation level during TOF stimulation was 0.9% (+1.6 SD, *p* < 0.05) (Figure 4).

### Time to Recovery From Neuromuscular Block

TOF monitoring in individual infants at the ulnar nerve showed that adductor pollicis (thumb) twitch can be reproducibly measured by visual observation and accelerometry.

#### Rocuronium Bolus Administration

**Figure 5:**
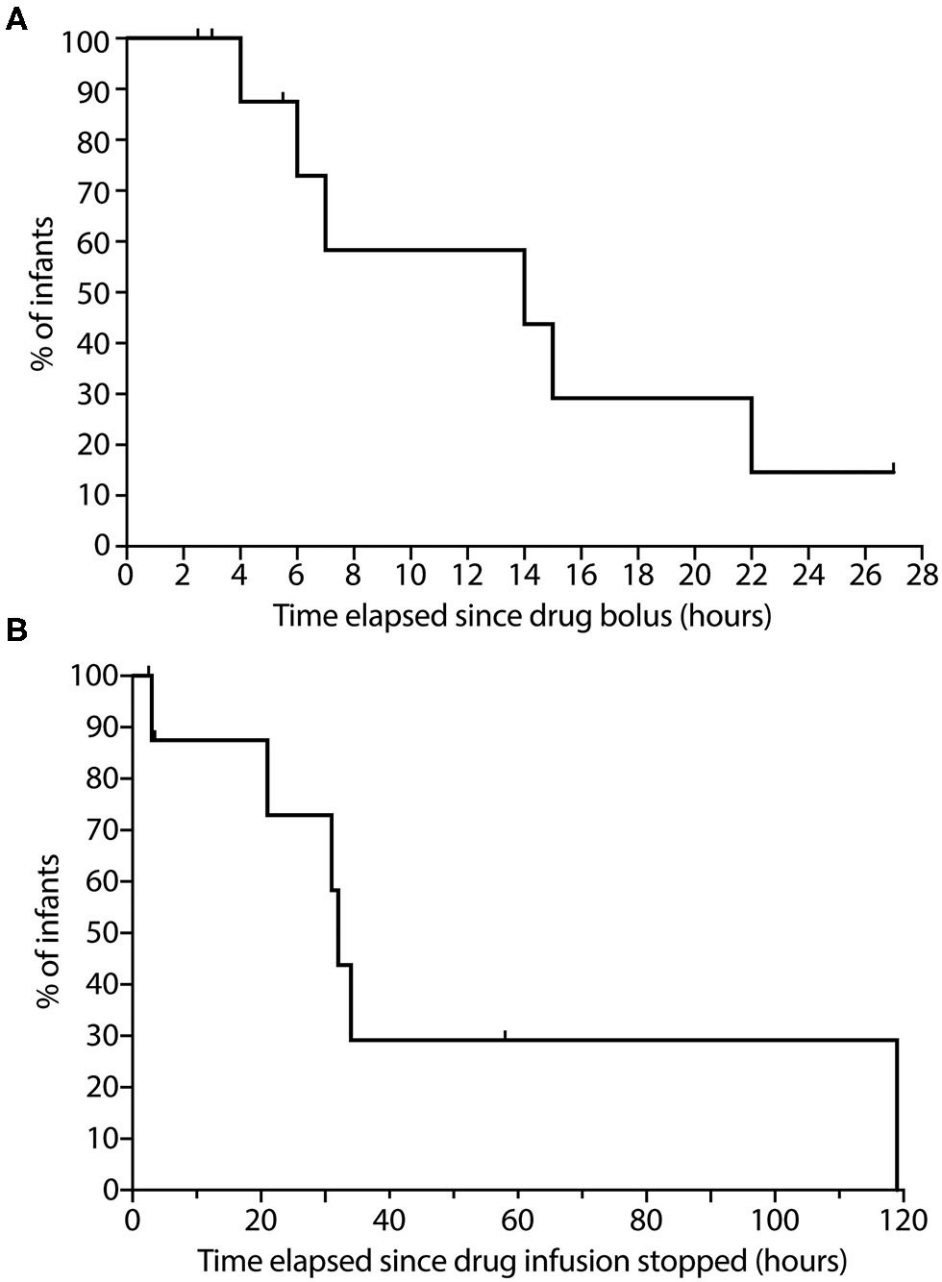
Time course to neuromuscular blockade recovery in infants following. **(A)** Rocuronium bolus dosing; and **(B)** Continuous vecuronium infusion. **(A)** The percentage of infants following rocuronium bolus dosing with TOF ratio <70% is displayed on the graph (*n* = 10). Data were collected up to 27 h of recovery from neuromuscular block. **(B)** The percentage of infants following cessation of continuous vecuronium infusion with TOF ratio <70% is displayed on the graph (*n* = 9). Data were collected up to 119 h of recovery from neuromuscular block. Ticks represent tested infants whose TOF ratio remained <70%. TOF, Train-of-Four.

Of the 10 patients in this group, we were able to capture data on all at some point in their NMB recovery, specifically 6 patients crossing the 70% threshold, 4 crossing the 80% threshold and 2 crossing the 90% threshold. For our primary outcome, the median time to recover to a TOF ratio above 70% was 14 h with a range of 4–22 h (Figure 5). For our secondary analysis, time to recover to a TOF ratio above 80% ranged between 6 and 22 h and for TOF ratio above 90% were 7–19 h.

#### Cessation of Vecuronium Infusion

Of the 9 patients in this group, we were able to record data on all during portions in their NMB recovery, specifically 6 patients crossing the 70% threshold, 4 crossing 80% threshold and 3 crossing the 90% threshold. For our primary analysis, median time to recovery of TOF ratio above 70% was 34 h with a range of 3–119 h (Figure 5). For our secondary analysis, time for reaching TOF ratio above 80% ranged between 2 and 119 h, and for TOF ratio above 90% ranged between 21 and 119 h. One patient in this group exhibited prolonged residual paralysis lasting to between 76 and 119 h (~3–5 days). This patient received vecuronium infusion for 8 days starting shortly after birth and exhibited generalized edema along with acute renal injury.

## Discussion

This pilot study has shown that: (1) Most infants had a twitch response as they recovered from NMB; (2) Median recovery times to TOF ratio >70% were 14 h following a large dose of a rocuronium bolus and 34 h following cessation of continuous vecuronium infusion; (3) Stimulus threshold was slightly lower for visual thumb twitch compared to accelerometry; and (4) No clinical meaningful change in heart rate and oxygen saturation was observed during TOF stimulation.

This pilot study shows that use of accelerometry and peripheral nerve stimulation elicited by the StimPod TOF device is technically feasible. However, a slightly higher stimulus amplitude threshold was required to elicit accelerometry output than for visual thumb twitch. Similar results were found in earlier research, where supramaximal stimulus amplitude as high as 60 mA was required to elicit accelerometry output (12). Though counterintuitive, these amplitudes are similar to those required for adults (13). One possible explanation might be mechanical. For smaller infants with partial degrees of NMB and with lower stimulus currents (e.g., 15–20 mA), muscle contraction may be visible, but the force generated may be less than the weight of the transducer, resulting in failure to accelerate the transducer (analogous to an isometric contraction). In these cases, increasing the stimulus amplitude further results in sufficient force generation to overcome the weight of the transducer and produce a measurable acceleration. Of note, the Stimpod 450 ms user manual did not include any age or size limitation. This inertial effect may explain the slight negative correlation between the bodyweight of the infant and the accelerometry stimulus threshold (Figure 3). A higher body weight correlates with higher muscle mass, including the thenar muscles; this additional strength may be more able to overcome the weight of the transducer.

In addition to the accelerometry performed on infants after cessation of continuous Vecuronium infusions, we also performed testing on 5 infants during continuous Vecuronium infusion at rates between 0.1 and 0.11 mg/kg/h. None of these infants demonstrated a visual thumb twitch or measurable acceleration. A reasonable interpretation is that the degree of NMB produced by vecuronium infusions at these rates may be greater than necessary to achieve adequate suppression of motion and tolerance of mechanical ventilation in infants and may result in prolonged recovery and other possible sequelae compared to lower infusion rates. The use of peripheral nerve stimulation with TOF device may be useful for individualized titration of infusion rates or for future studies of NMBA dose response, and may complement information derived from the common clinical practice of “vecuronium holidays” (13, 14).

Three patients required re-intubation within 24 h of planned tracheal extubation. All three had a TOF ratio <70% prior to their extubation. Five patients required non-invasive positive pressure respiratory support (i.e., Continuous Positive Airway Pressure, or Non-Invasive Positive Pressure Ventilation) in order to remain extubated and their TOF ratios were between 70 and 80%. Even though the association between residual NMB and respiratory failure in the adult population is well-established, a larger clinical outcome study is needed to demonstrate a possible association between residual NMB and respiratory complications among infants after surgery or in the NICU (1, 15, 16). Clinicians visual detection of twitch fade is often inaccurate at TOF ratios >40%, leading to a false assumption of adequate neuromuscular recovery (17).

The primary factors leading to slow and incomplete NMB recovery following NMBA administration among NICU infants could include hepatic and renal immaturity, acid-base or electrolyte derangement, or accumulation in an expanded extracellular fluid volume. In the case of vecuronium, slow clearance of a pharmacologically active metabolite may also contribute to prolonged weakness, especially in the setting of impaired or immature renal clearance (18). These findings might also represent biologic changes in neuromuscular junction function following NMBA exposure (16). From a neurodevelopmental perspective, neuromuscular transmission is immature in neonates and small infants until 2-months of age. Therefore, it is entirely plausible that some infants may have a reduced TOF ratio without muscle relaxation (19). In order to identify if these factors play a significant role in the variability of NMB recovery among neonates, future combined pharmacokinetic—pharmacodynamic studies are needed.

### Limitations

In addition to the relatively small sample size of both the rocuronium bolus dosing group and the prolonged vecuronium infusion group, this pilot study was limited by challenges in capturing adequate NMB recovery (TOF ratio >90%) in every infant due to various clinical circumstances (e.g., earlier than expected cessation of NMB and extubation, clinical instability). Despite these limitations, we were able to demonstrate clinical feasibility of these measurements and were able to provide pilot information suggesting that under common conditions of clinical practice, recovery from NMB is slow and variable in the NICU. This variability supports the need for frequent NMB monitoring until adequate recovery is achieved.

Neuromuscular reversal agents are used very commonly in the OR, but rarely in the NICU. Future studies may define the risk-benefit ratio of selective use of neuromuscular reversal for infants in the NICU.

In conclusion, this study demonstrates the feasibility of objective monitoring of NMB depth level in the NICU and suggests considerable variability in time course of NMB time recovery, particularly following vecuronium infusions. Future studies may define the relative contributions to this variability from pharmacokinetics, neuromuscular development, or specific aspects of newborn critical illness *per se*. If use of TOF measurements becomes widespread, a neonatal sized accelerometer might be beneficial. Ensuring acceptable NMB recovery may help reduce the contribution of residual paralysis to cases of failed extubation. Larger clinical outcome studies are recommended to assess whether or not TOF monitoring should become a standard practice for NICU patients, and whether its use can contribute to clinical benefits, including better individualized dose titration, reduced frequency of failed extubation, or reduced neuromuscular sequelae of critical illness in the NICU.

## Data Availability Statement

All datasets generated for this study are included in the Supplementary Material.

## Ethics Statement

The studies involving human participants were reviewed and approved by Boston Children's Hospital Institutional Review Board. Written informed consent to participate in this study was provided by the participants' legal guardian/next of kin.

## Author Contributions

OS, LC, and CB defined the research idea and designed the study method. OS wrote the initial report. OS, LC, AK, and EU contributed to the research data collection and design of data collection tools. OS and AH were responsible for the clinical care of the participants. OS, LC, and CB performed the statistical analysis. OS, CB, LC, AH, EU, and AK reviewed and edited the manuscript. All authors have read and approved the final version of this manuscript.

## Conflict of Interest

The authors declare that the research was conducted in the absence of any commercial or financial relationships that could be construed as a potential conflict of interest.

## Supplementary Material

Supplementary File 1 The datasets generated and analyzed in this study.
